# Supplementary material for: IL-10 Gene Polymorphisms and IL-10 Serum Levels in Patients with Multiple Sclerosis in Lithuania
Source: Brain Sci. 2022 Jun 18;12(6):800. doi: 10.3390/brainsci12060800 (PMC9221209; doi:10.3390/brainsci12060800)
Supplement: Supplementary file 1 [file brainsci-12-00800-s001.zip › brainsci-1742965-supplementary.pdf]

Supplementary Materials:

Table S1. Binary logistic regression analysis of *IL-10* rs1800871, rs1800872, and rs1800896 genotypes.

Genetic Model	Genotype/Allele	OR (95% CI)	*p*-Value	AIC
		***IL-10* (rs1800871):**		
Codominant	AG vs. GG	0.755 (0.352–1.622)	0.472	167.376
	AA vs. GG	1.091 (0.0.206–5.787)	0.919	
Dominant	AG+AA vs. GG	0.790 (0.379–1.646)	0.529	165.553
Recessive	AA vs. GG+AG	1.216 (0.235–6.284)	0.816	165.897
Overdominant	AG vs. AA+GG	0.750 (0.353–1.592)	0.454	165.386
Additive	G	0.872 (0.472–1.610)	0.661	165.757
		***IL-10* (rs1800872):**		
Codominant	TG vs. GG	0.923 (0.433–1.970)	0.836	167.495
	TT vs. GG	1.600 (0.332–7.717)	0.558	
Dominant	TG+TT vs. GG	0.993 (0.480–2.053)	0.985	165.950
Recessive	TT vs. GG + TG	1.653 (0.354–7.732)	0.523	165.537
Overdominant	TG vs. GG + TT	0.882 (0.420–1.854)	0.741	165.841
Additive	G	1.074 (0.592–1.946)	0.814	165.896
		***IL-10* (rs1800896):**		
Codominant	TC vs. TT	1.294 (0.796–2.103)	0.299	490.351
	CC vs. TT	1.562 (0.866–2.820)	0.138	
Dominant	TC + CC vs. TT	1.372 (0.870–2.161)	0.173	489.808
Recessive	CC vs. TT + TC	1.340 (0.803–2.235)	0.263	489.437
Overdominant	TC vs. TT + CC	1.082 (0.711–1.647)	0.713	488.549
Additive	T	1.254 (0.936–1.680)	0.130	488.377

p-value—significance level (statistically significant when $p < 0.05$); OR—odds ratio; AIK—Akaike information criterion. *p*-values, if statistically significant, are marked in bold.

Table S2. Binary logistic regression analysis of *IL-10* rs1800871, rs1800872, and rs1800896 genotypes in males.

Genetic Model	Genotype/Allele	OR (95% CI)	*p*-Value	AIC
		***IL-10* (rs1800871):**		
Codominant	AG vs. GG	0.755 (0.352–1.622)	0.472	167.376
	AA vs. GG	1.091 (0.206–5.787)	0.919	
Dominant	AG + AA vs. GG	0.790 (0.379–1.646)	0.529	165.553
Recessive	AA vs. GG + AG	1.216 (0.235–6.284)	0.816	165.897
Overdominant	AG vs. AA + GG	0.750 (0.353–1.592)	0.454	165.386
Additive	G	0.872 (0.472–1.610)	0.661	165.757
		***IL-10* (rs1800872):**		
Codominant	TG vs. GG	0.923 (0.433–1.970)	0.836	167.495
	TT vs. GG	1.600 (0.332–7.717)	0.558	
Dominant	TG + TT vs. GG	0.993 (0.480–2.053)	0.985	165.950
Recessive	TT vs. GG + TG	1.653 (0.354–7.732)	0.523	165.537
Overdominant	TG vs. GG + TT	0.882 (0.420–1.854)	0.741	165.841
Additive	G	1.074 (0.592–1.946)	0.814	165.896
		***IL-10* (rs1800896):**		
Codominant	TC vs. TT	0.958 (0.410–2.237)	0.920	167.924
	CC vs. TT	1.029 (0.380–2.789)	0.955	

Dominant	TC + CC vs. TT	0.981 (0.444–2.165)	0.962	165.949
Recessive	CC vs. TT + TC	1.057 (0.452–2.472)	0.898	165.934
Overdominant	TC vs. TT + CC	0.945 (0.459–1.949)	0.879	165.928
Additive	T	1.011 (0.615–1.664)	0.964	165.949

Table S3. Binary logistic regression analysis of *IL-10* rs1800871, rs1800872, and rs1800896 genotypes in females.

***IL-10* (rs1800871):**				
**Genetic Model**	**Genotype/Allele**	**OR (95% CI)**	***p*-Value**	**AIC**
Codominant	AG vs. GG	0.915 (0.532–1.574)	0.749	325.689
	AA vs. GG	0.439 (0.116–1.668)	0.227	
Dominant	AG + AA vs. GG	0.843 (0.499–1.424)	0.523	324.923
Recessive	AA vs. GG + AG	0.455 (0.122–1.698)	0.241	323.792
Overdominant	AG vs. AA + GG	0.975 (0.571–1.662)	0.925	325.323
Additive	G	0.803 (0.517–1.246)	0.327	324.359
***IL-10* (rs1800872):**				
Codominant	TG vs. GG	0.900 (0.522–1.552)	0.705	323.648
	TT vs. GG	0.437 (0.115–1.658)	0.224	
Dominant	TG + TT vs. GG	0.829 (0.490–1.402)	0.484	324.841
Recessive	TT vs. GG + TG	0.455 (0.122–1.698)	0.241	323.792
Overdominant	TG vs. GG + TT	0.958 (0.560–1.639)	0.876	325.307
Additive	G	0.793 (0.510–1.233)	0.304	324.257
***IL-10* (rs1800896):**				
Codominant	TC vs. TT	1.479 (0.813–2.688)	0.200	324.132
	CC vs. TT	1.895 (0.906–3.965)	0.089	
Dominant	TC + CC vs. TT	1.592 (0.909–2.789)	0.104	322.635
Recessive	CC vs. TT + TC	1.503 (0.791–2.858)	0.214	323.797
Overdominant	TC vs. TT + CC	1.159 (0.690–1.945)	0.578	325.021
Additive	T	1.387 (0.964–1.996)	0.078	322.202
